# Coupling Bulk Phase Separation of Disordered Proteins to Membrane Domain Formation in Molecular Simulations on a Bespoke Compute Fabric

**DOI:** 10.3390/membranes12010017

**Published:** 2021-12-23

**Authors:** Julian C. Shillcock, David B. Thomas, Jonathan R. Beaumont, Graeme M. Bragg, Mark L. Vousden, Andrew D. Brown

**Affiliations:** 1 Blue Brain Project and Laboratory of Molecular and Chemical Biology of Neurodegeneration, Ecole Polytechnique Fédérale de Lausanne, CH-1015 Lausanne, Switzerland; 2 Department of Electronics and Computer Science, University of Southampton, Highfield, Southampton SO17 1BJ, UK; D.B.Thomas@southampton.ac.uk (D.B.T.); gmb@ecs.soton.ac.uk (G.M.B.); M.Vousden@soton.ac.uk (M.L.V.); adb@ecs.soton.ac.uk (A.D.B.); 3 Department of Electronic Engineering, Imperial College London, London SW7 2AZ, UK; jonathan.r.beaumont@gmail.com

**Keywords:** membrane domains, intrinsically-disordered protein, phase separation, biomolecular condensate, coarse-grained simulation, event-based computing, hardware-accelerated simulation, application-specific hardware

## Abstract

Phospholipid membranes surround the cell and its internal organelles, and their multicomponent nature allows the formation of domains that are important in cellular signalling, the immune system, and bacterial infection. Cytoplasmic compartments are also created by the phase separation of intrinsically disordered proteins into biomolecular condensates. The ubiquity of lipid membranes and protein condensates raises the question of how three-dimensional droplets might interact with two-dimensional domains, and whether this coupling has physiological or pathological importance. Here, we explore the equilibrium morphologies of a dilute phase of a model disordered protein interacting with an ideal-mixing, two-component lipid membrane using coarse-grained molecular simulations. We find that the proteins can wet the membrane with and without domain formation, and form phase separated droplets bound to membrane domains. Results from much larger simulations performed on a novel non-von-Neumann compute architecture called POETS, which greatly accelerates their execution compared to conventional hardware, confirm the observations. Reducing the wall clock time for such simulations requires new architectures and computational techniques. We demonstrate here an inter-disciplinary approach that uses real-world biophysical questions to drive the development of new computing hardware and simulation algorithms.

## 1. Introduction

Soft surfaces are ubiquitous in living cells and are typified by phospholipid membranes that bound the cell and its internal organelles [1]. Membranes allow compositionally-distinct environments for incompatible biochemistry and provide specialized surfaces to mediate material and information transport. Although viewed originally as merely structural boundaries, they are now recognized as active participants in cellular dynamics [2,3]. They undergo morphological transitions between distinct shapes in response to weak external influences [4], and in turn exert forces on membrane-associated particles or materials [5]. Their multicomponent nature allows regulated demixing of a subset of their component lipids and proteins to assemble into localized domains to carry out transient functions [3,6]. Pathological domain formation occurs when bacteria such as *E. Coli* and Shigelle release nm-sized rigid toxin particles in order to infect the cell [7]. Toxin particles adsorb to the cellular plasma membrane, cluster into domains, and invaginate into the cell carrying their toxic payload that ultimately destroys the cell and aids the life cycle of the bacterium [8].

Another form of fluid interface that is increasingly recognized as crucial for compartmentalizing the cellular and nuclear cytoplasm is the surface of biomolecular condensates or membraneless organelles [9,10,11,12]. These are fluid droplets that form by liquid–liquid phase separation (LLPS) of a wide range of intrinsically-disordered proteins (IDPs) [13]. They have numerous functions in the cell, including regulation of biochemical reactions [14], sequestering stalled RNA translation in stress granules [15], chromatin organization in the nucleus [16], and organizing the presynaptic axon and postsynaptic density in neurons [17,18]. Unlike lipid membranes, the formation of biomolecular condensates (BCs) in vivo is usually reversible: The cell actively assembles them to perform a function and they subsequently melt away [19]. However, they can transition irreversibly into pathological rigid states in experiments [20,21,22], a transition that is hypothesized to be important for neurodegenerative diseases such as Alzheimer’s, Parkinson’s and Huntington’s disease [23]. Evidence is emerging that they are implicated in the dysregulation of cellular processes occurring in cancer [24,25,26].

The cellular cytoplasm is a crowded place: It is estimated that nowhere in a cell is more than 50 nm from a lipid membrane [1]. The combination of two-dimensional fluid lipid membranes, which can be relatively easily deformed, and three-dimensional fluid protein droplets could theoretically lead to a variety of morphologies [4,27,28,29]. Membranes regulate the formation of BCs that help assemble protein signaling clusters [30,31,32], and mediate immune cell signalling [33,34,35]. Recent experiments show that phase separation of T cell proteins at the surface of giant unilamellar lipid vesicles drives lipid domain formation that in turn is able to recruit additional proteins to the domains [36]. This provides a starting point for rational design of artificial organelles [37].

In this work, we qualitatively explored the equilibrium morphologies of a dilute phase of self-associating model IDPs near a well-mixed, two-component lipid bilayer using the coarse-grained, explicit-solvent simulation technique of dissipative particle dynamics (DPD) [38,39,40], which is commonly used for simulating membranes [41,42], and complex fluids [43]. In our model, the two lipid species have identical molecular structure and interactions with each other, so that only their interaction with the IDPs can drive domain formation. The IDPs are modeled as soluble telechelic polymers (linear molecules with self-associating endcaps [44]). We first investigated how the IDPs organize the minority phase lipid by direct interaction with their headgroups, to simulate wetting phenomena. We then extended our exploration by attaching a polymeric linker to the minority phase lipid headgroups to better mimic the flexible cytoplasmic tails of transmembrane signalling proteins [32,35]. The linkers contain a linear sequence of sticky and inert domains, and we explored the morphological outcome of the system when the IDP endcaps are attracted to the sticky domains. 

**Figure 1:**
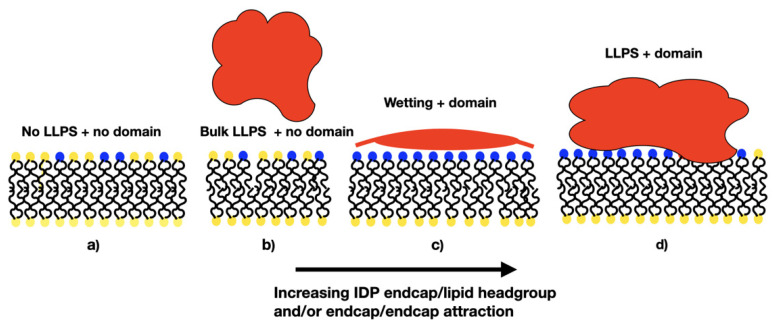
Cartoon showing potential morphologies of the bulk IDPs and two-component lipid membrane as the relative interactions between the IDPs (red) and the minority lipid headgroups (blue) are varied. (**a**) IDPs dispersed in the solvent; (**b**) high self-attraction; (**c**)thin wetting layer; (**d**) minority phase lipid domain.

We a priori envisaged several morphological outcomes for the IDP/membrane system as the relative strengths of the IDP self-attraction and IDP-lipid/linker attraction are varied, and these are illustrated in Figure 1.

When both interactions are weak, the membrane is well mixed and the IDPs are dispersed in the solvent (a, IDPs not shown). In the absence of an attraction of the IDPs for the minority phase lipids, we expected the membrane to be well mixed, and the IDPs dispersed in the bulk solvent if their self-attraction is low (as in a), or phase separated, if their self-attraction is high (b). Increasing the attraction of the IDPs for the lipid headgroups leads to a morphology that is controlled by their self-attraction: if this is low, they may form a thin wetting layer (c), while a strong self-attraction may lead to a phase separated droplet adhering to a minority phase lipid domain (d). We observed all these results in the simulations, indicating that the equilibrium states of the membrane and IDPs are accessible to DPD simulations. Theoretical calculations have predicted a range of wetting states resulting from the adsorption of polymeric solute molecules on a membrane surface. In particular, it was found that surface phase transitions can occur under conditions in which bulk phase separation is impossible at any solute concentration [27]. Monte Carlo simulations have been used to study the cooperativity of receptor-ligand binding and domain formation in membrane binding [45]. But a molecular-scale picture of the structural organization of membrane-associated BCs is still missing.

Although our system contains only three molecular species (not including the solvent), a large number of parameters must be specified to define its state. Additionally, the simulated system is relatively small (~10–100 nanometres in linear dimension), and we may legitimately ask: What are the prospects of improving its biological fidelity? In considering this question, we encounter the barrier of the rapidly increasing computational cost of simulating a system of increasing size while retaining near-molecular resolution. 

Biological membranes evolve on multiple timescales that are hard to span even with coarse-grained simulation techniques [46,47,48,49]. The resources needed to simulate a membrane of linear dimension L on a single compute core scale at least as L^5^, allowing for the spatial increase and time for diffusive processes to propagate, so it is challenging to extend such simulations to near-cell scale problems (~microns). The Folding@home consortium recently harnessed more than one million devices across the world to explore conformational transitions of the SARS-CoV-2 spike protein with an effective performance of 1.01 exaflops [50]. Even this extraordinary power is not sufficient to simulate the interaction of, for example, a complete virus particle with the host cell membrane [49].

This is not a new problem. Conventional parallel programming paradigms using, e.g., the message passing interface (MPI) system, increase the simulated volume by dedicating many compute cores to one simulation [51]. However, they are limited by the requirement that the compute cost per core must exceed the messaging cost, which necessitates a large number of particles to be assigned to each core. This limitation is not removed by adding more cores, but it can be eliminated by the elegant scheme we describe below, and which we have used to carry out large-scale simulations to validate our main results.

Since 2016, the Engineering and Physical Sciences Research Council of the UK has funded a project called POETS—Partially-Ordered Event-Triggered Systems (POETS)—that applies the non-von-Neumann computer architecture called event-based computing to large-scale computational problems [52]. POETS is not a general-purpose computer: Each application must be transformed into a graph in order to run on POETS, and reimplemented in the POETS domain specific language. We took the open source DPD code Osprey-DPD [53], converted it to run in POETS, and used it to explore the larger system size presented here. POETS-DPD allowed us to simulate larger systems at a reduced cost—both in terms of wall clock time and electricity consumed—compared to conventional CPU/GPU hardware [54].

In POETS-DPD, the simulation volume is divided into a grid of cells similar to that used in conventional MPI-based parallel schemes. Crucially, in contrast to MPI-based simulations, each grid cell contains only a few particles and is assigned to its own compute core, so each grid cell’s computing step is very fast. Messaging between cores is executed in hardware and is also fast. Because the limitation of compute cost >> messaging cost has been removed, the system as a whole can be scaled to any volume by simply adding more cores, and it exhibits almost perfect weak scaling.

We showcase in this special issue results obtained with conventional DPD and POETS-DPD, and demonstrate that event-based computing opens up exciting pathways to near cell-scale computational exploration. We use an inter-disciplinary approach that combines the development of new hardware and simulation algorithms on the bespoke computational hardware with the real-world biophysical problem of predicting the influence of protein phase separation on membrane organization. The POETS-DPD hardware and software is still under development, but the initial version used here provides functionally correct answers at speeds which are substantially faster than existing systems, and allows us to validate the dynamical phenomena we observe between small and large systems over long timescales.

The principal contributions of this paper are two-fold:

1. We present a simulation-based exploration of the morphological outcomes resulting from the 3D phase separation of intrinsically-disordered proteins near a 2D lipid membrane;
2. We describe the novel POETS compute architecture and the algorithm used in POETS-DPD, and demonstrate the power of this approach by simulating selected systems at a fraction of the wall clock time: a 14 cpu-day conventional DPD simulation is completed by POETS-DPD in 8 h, a speedup of more than a factor of 40.

The remainder of the paper is organized as follows. In Section 2, we briefly describe the dissipative particle dynamics simulation technique and the event-based hardware and POETS-DPD implementation, referring the reader to the literature for more details. Section 3 presents our main results on the equilibrium morphologies observed in the simulations. Section 4 discusses the powerful advantage of POETS-DPD by highlighting much larger systems. Finally, in Section 5, we illuminate the path ahead for using massively parallel POETS-DPD to explore near-cell scale dynamic phenomena.

## 2. Materials and Methods

### 2.1. Dissipative Particle Dynamics Simulations

Dissipative particle dynamics (DPD) is a coarse-grained, explicit-solvent form of molecular dynamics originally designed to simulate the hydrodynamic behavior of complex fluids [38,39,40]. In DPD, atoms and atomic groups are grouped into beads, of mass m, which interact via three non-bonded, short-ranged (vanish beyond a cut-off distance *d_0_*), soft, momentum-conserving forces. A conservative force between each bead type pair (characterized by a strength parameter $a_{ij}$ for beads of type *i* and *j*) gives beads a chemical identity, such as hydrophobic oil particles or hydrophilic amino acid residues. Additionally, a dissipative force (characterized by the parameter $\gamma_{ij}$) and random force (whose strength $\sigma_{ij}$ is related to the dissipative force parameter by the fluctuation-dissipation theorem $\sigma_{ij}^{2}=2\gamma_{ij}k_{B}T$) provide a thermostat that keeps the system temperature $k_{B}T$ constant. More details of the force field are given in previous work [55]. Beads are connected into molecules with Hookean springs, and a bending stiffness may be applied via an additional bond angle-dependent potential. Once the forces are specified for all bead and molecule types in a simulation, the Newtonian equations of motion for the beads are integrated using a modified Velocity Verlet scheme. Because the non-bonded forces are soft, the integration scheme is able to use a larger time-step than is possible in atomistic molecular dynamics, and the grouping of several atomic groups into each bead reduces the number of degrees of freedom needing to be integrated. These two factors make DPD several orders of magnitude faster than traditional atomistic and coarse-grained molecular dynamics. 

**Figure 2:**
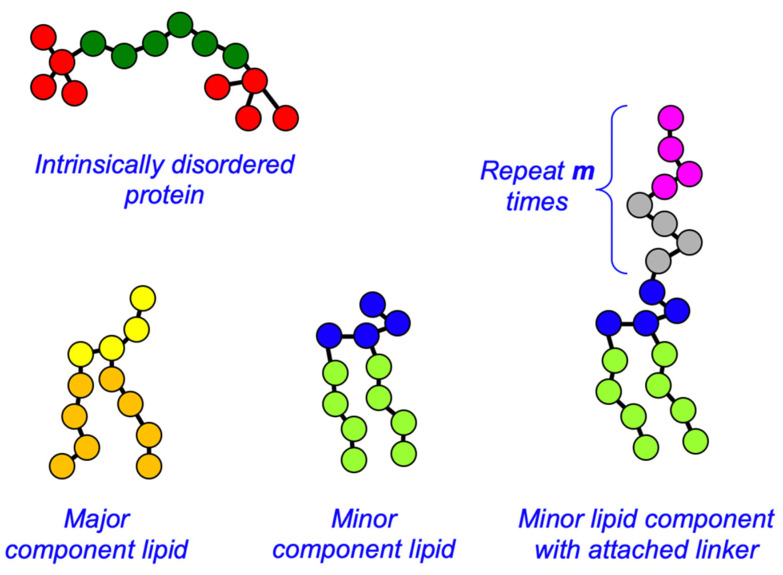
Cartoon of the molecular shape of the IDPs and lipid species. The conservative interactions for all bead types are given in Table 1. Note that the head beads and tail beads of the two lipid species are colored differently for clarity but are otherwise the same in the simulations. The number of linker elements can be varied, but is fixed at m = 8 for simplicity. The number of backbone beads in the IDPs is shown as six, but is varied as part of the investigation.

DPD has been extensively used to simulate lipid membranes [41,56], domain formation in two-component membranes [57,58], nanoparticles interacting with membranes [59,60], and many other soft matter systems [43]. We adopt here the model of Grafmüller et al. for the lipid molecules [61], and their molecular shape is shown in Figure 2. Briefly, each lipid has a headgroup composed of four hydrophilic beads (H_A_ for the major species, H_B_ for the minor species), and two tails each containing four hydrophobic beads (T_A_ for the major species, T_B_ for the minor species). The tails are connected to the final two adjacent head beads as shown in Figure 2. The IDPs are modeled as linear, semi-flexible polymers (backbone beads of type B) with self-associating endcaps containing four beads (bead type E) [55]. Water was represented by a single bead W. Both backbone and endcap beads in the IDPs are hydrophilic, and it is only the self-attraction of their endcaps that drives their phase separation. The full set of DPD conservative force parameters is given in Table 1. The two parameters $a_{EE}$ and $a_{EM}$ set the attraction between the IDP endcaps and themselves and the minority lipid headgroups, respectively, and are varied systematically in Section 3.1. The parameter $a_{ES}$ sets the attraction of the IDP endcaps and the sticky domains in the linker attached to the minority phase lipid in Section 3.4. The dissipative force parameter $\gamma_{ij}$ for all beads was 4.5 (in units of $\sqrt{mk_{B}T/d_{0}^{2}}$). Beads were bonded into molecules using Hookean springs with potential parameters of 128 and 0.5 (in units of $k_{B}T/d_{0}^{2}$ and $d_{0}$ respectively). A three-body bending potential of the form $k_{3}\;\left(1-cos\left(\varphi -\varphi_{0}\right)\right)$ was applied to adjacent triples of backbone beads (B) in the IDPs, and the linker beads L and S when they were present, with parameters $k_{3}=5$ $k_{B}T$ and $\varphi_{0}=0$. A similar potential was applied to the lipid tails with parameters $k_{3}=15$ $k_{B}T$ and $\varphi_{0}=0,$. Further details of the simulation technique are given in the literature [40,55,61].

All simulations took place in a box of size 40 × 40 × 48 (*d_0_*)^3^ unless otherwise stated and periodic boundary conditions were applied in all three dimensions. A single planar membrane was pre-assembled with its normal along the long axis, and the minority phase lipids were randomly distributed in the upper monolayer only to localize IDP/membrane interactions to one side. A given number of IDPs were dispersed randomly throughout the remaining space and the simulation box was filled with solvent beads to the average bead density $\rho d_{0}^{3}=3.$ The DPD length scale is set to $d_{0}=1\;\mathrm{nm}$ from the size of the lipid headgroups [41]. We set the reduced system temperature to $k_{B}T=1$ [40]. Each simulation was run for 3 million time-steps unless otherwise stated using an integration step size of 0.02 τ, where $\tau =\sqrt{md_{0}^{2}/k_{B}T}$ is the DPD timescale. Because we are interested in equilibrium properties, we did not attempt to fix the simulation timescale more precisely.

Results in the 40 × 40 × 48 (*d_0_*)^3^ simulation box are generated using the open-source, single-thread DPD code OSPREY-DPD [53]. Simulations in the larger box 100 × 100 × 48 (*d_0_*)^3^ are performed using POETS-DPD as described next.

### 2.2. Massively Parallel Event-Based Simulations

The larger simulations in this work were performed on a new computer technology called POETS—Partially Ordered Event-triggered Systems [52]. POETS represents computational problems as a graph: vertices in the graph are independent computational threads; and edges between vertices represent channels that transport messages between threads. The abstract, arbitrary computational graph is mapped onto a fixed hardware network of RISC-V processors [62], which provides tens of thousands of hardware threads in the space and power envelope that would usually support only a few hundred conventional x86 threads. POETS is an ongoing research project (https://www.poets-project.org; accessed on 18 December 2021) but preliminary results show that it is highly suited to DPD simulations [54]. We used it both as an efficient way to produce the large-scale results presented here, and to evaluate its potential for production use in mesoscale research and simulation.

POETS is not a general-purpose computer. Each application must be transformed into a graph in order to run on POETS, and reimplemented in the POETS domain specific language. For our case of DPD simulations, we follow the same discretization of space approach used to parallelize DPD for the MPI framework. The simulation volume is spatially subdivided into a cubic array of unit cells for which the beads in each cell are managed by a single computational thread. Each cell becomes a vertex in the graph, and each vertex has edges to its 26 neighboring cells to represent information flows within each simulated time-step. A key point of POETS messaging is that it is very fast and consumes few cycles: sending a message is just one machine instruction, with message latencies ranging from 1–10 micro-seconds, depending on the physical distance between cores. This contrasts with MPI messages that are typically very slow compared to a local function call, requiring hundreds of instructions to prepare, and with latencies ranging from 10–1000 micro-seconds, depending on the message size.

**Figure 3:**
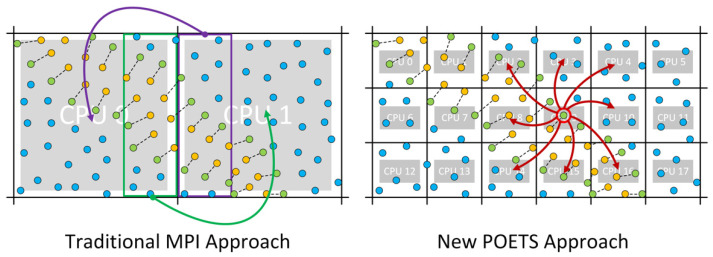
Contrast between the traditional MPI-based parallelization approach (left panel) in which each CPU owns a volume of space containing hundreds or thousands of beads, and the POETS approach (right panel) where space is sub-divided down to unit-volume cells containing only 3 or 4 beads, each of which is managed by one light-weight CPU.

A big algorithmic difference between DPD on POETS and MPI is that the volume of space managed by a cell in the POETS implementation is comparable to the DPD non-bonded cut-off distance (*d_0_*), so each cell contains only a few particles. In comparison, an MPI-enabled DPD must use much larger cells containing thousands of beads to ensure that the messaging cost is a small fraction of the total runtime. The difference between these two approaches is shown graphically in Figure 3. Effectively, because messaging in POETS is so cheap, we are able to use very large numbers of light-weight CPUs with each managing only a few beads. 

Once ownership of all the unit cells in the simulation space has been assigned to physical POETS CPUs, the DPD algorithm executes in a highly concurrent fashion, with each thread operating independently and synchronized only by the messages containing beads. We only sketch the main ideas here, in order to give a sense of how it works and refer the read to the literature for more details [54]. POET-DPD executes in in two phases:

1. **Force calculation**
  a **Sharing**: Broadcast each resident bead position and velocity to neighbors;
  b **Integration**: For each received bead, calculate non-bonded DPD and Hookean bond interactions with resident cells;
  c **AngleForces**: Once head and tail beads for an angle bond are received by middle bead, calculate angle forces and broadcast to neighbors (which must include the owner of the angle bond’s head and tail;
  d **AngleAddition**: If an angle force is received ***and*** this cell contains the related head or tail bead, apply angle-bond forces to that head/tail.
2. **Bead movement**
  a **Movement**: Apply equations of motion to all beads resident in the cell;
  b **BeadExit**: If any bead leaves the cell, broadcast it to neighbors and remove from resident set;
  c **BeadEntrance**: If a bead is received from a neighbor ***and*** its new position is in the current cell, add it to the resident set.
3. Go back to step 1 for next step of simulation

This is a very high-level overview, but it highlights some of the important computational characteristics:

**Concurrent**: Each thread is running independently, so this approach is concurrent, where lots of threads do different things with loose synchronization, rather than parallel, where lots of threads to the same thing with tight synchronization.

**Fine-grained**: The simulation is decomposed into one computational thread per unit-volume cell, with each thread potentially only handling 3 or 4 beads, and resulting in thousands or millions of concurrent compute threads.

**Event-triggered**: Messages trigger computation, rather than computation being used to schedule messages. Within each phase, the sending and receiving of messages can be interleaved, overlapping compute and communication.

**Broadcasts**: Rather than aiming messages only at the correct receiver, we broadcast messages to all possible receivers. This might seem counter-intuitive, but due to the tight binding between processors and network in POETS this is very efficient.

**Single algorithm**: All calculations, including Hookean and angle bonds, are integrated into the same algorithm on the same compute thread, and handled concurrently with DPD force calculation.

There is obviously a lot more computational detail in precisely how this is implemented, not least because the experimental goals of the paper were used to drive development of the algorithms and methods. This meant that we had to handle the edge-cases needed in practical simulations, rather than just assuming-away the difficult parts in the pursuit of headline performance figures that do not translate to real-world performance.

For example, the need to handle angle bonds completely changed the way that we handled bonds within the algorithm. Our original algorithmic approach used a faster method with fewer algorithmic steps, but ultimately could only handle Hookean bonds efficiently. Though it was very fast, this was for a problem which is less useful in practice for end-users. By having computer scientists, hardware engineers, and bio-physics end-users working together we tried to avoid that trap, and tackle the more difficult but useful problems head on.

## 3. Results

### 3.1. Influence of the IDP-Membrane Affinity on the Equilibrium Morphology

We were interested in how compositionally-distinct domains in a multicomponent membrane may be induced by the nearby bulk phase separation of intrinsically-disordered proteins. As stated, the problem has too many parameters to be efficiently presented, and it is unfeasible to exhaustively explore its high-dimensional parameter space. We therefore studied the simplest system that may exhibit interesting behavior—a two-component membrane and a single type of IDP—and qualitatively enumerate its equilibrium states. The membrane contains a major lipid species and a smaller fraction of a minor lipid species, and we fixed their number fractions to give 1989 and 397 major and minor lipid molecules, respectively, corresponding to a minor fraction of 0.166. This ensures that a domain (if formed) is much larger than the lipid molecules but smaller than the membrane area, thereby reducing the effects of the boundary conditions. The lipid molecules possess identical structures and interactions and are well mixed in the absence of any influence of the IDPs. We initially fixed the backbone length of the IDPs to 6 beads and refer to them by their backbone length in an obvious notation as B_6_. We started with 397 B_6_ molecules in the bulk phase. Note that the IDP backbone and endcap beads are hydrophilic, and only their endcap self-attraction drives their phase separation. We varied the IDP self-attraction and their affinity to the minority phase lipid headgroups by changing the conservative DPD interaction parameters $a_{EE}$, $a_{EM}$ respectively. We defined dimensionless parameters to quantify the IDP endcap–endcap attraction ($\varepsilon_{EE}$), and the endcap-minority lipid headgroup ($\varepsilon_{EM}$) attraction by:

$$\varepsilon_{EE}=\left(a_{WE}-a_{EE}\right)/a_{WE}\;$$

$$\varepsilon_{EM}=\left(a_{WE}-a_{EM}\right)/a_{WE}$$

where $a_{WE}$ is the DPD conservative interaction parameter for the water/IDP-endcap interaction. We explored the range $0\;\leq \;\varepsilon_{ij}\;\leq 1$, for which 0 corresponds to no net attraction between bead types i, j (compared to their interaction with the solvent beads) and 1 to a very strong attraction.

**Figure 4:**
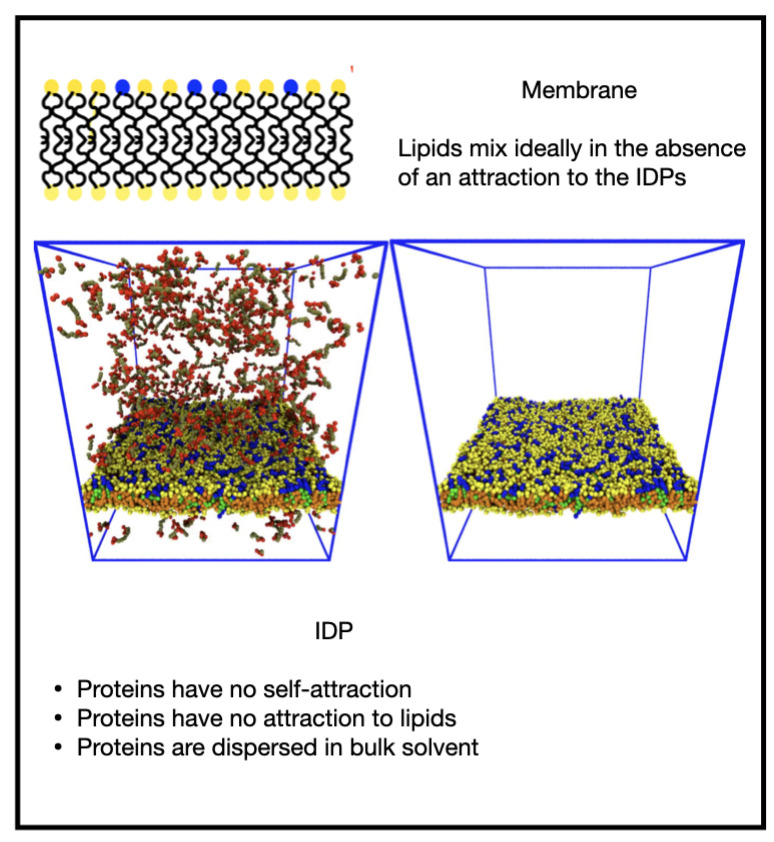
Equilibrium state of the system when both the IDP endcap self-attraction and attraction to the minority phase lipid headgroups are zero (solvent particles are invisible for clarity).

When there was no attraction of the IDPs for themselves nor the membrane, $\varepsilon_{EE}=\varepsilon_{EM}=0$, they remained dispersed in the solvent and the membrane was well mixed (Figure 4, Supplementary Video S1). In the following figures, the left panel shows the whole system while the right panel shows just the membrane for clarity.

**Figure 5:**
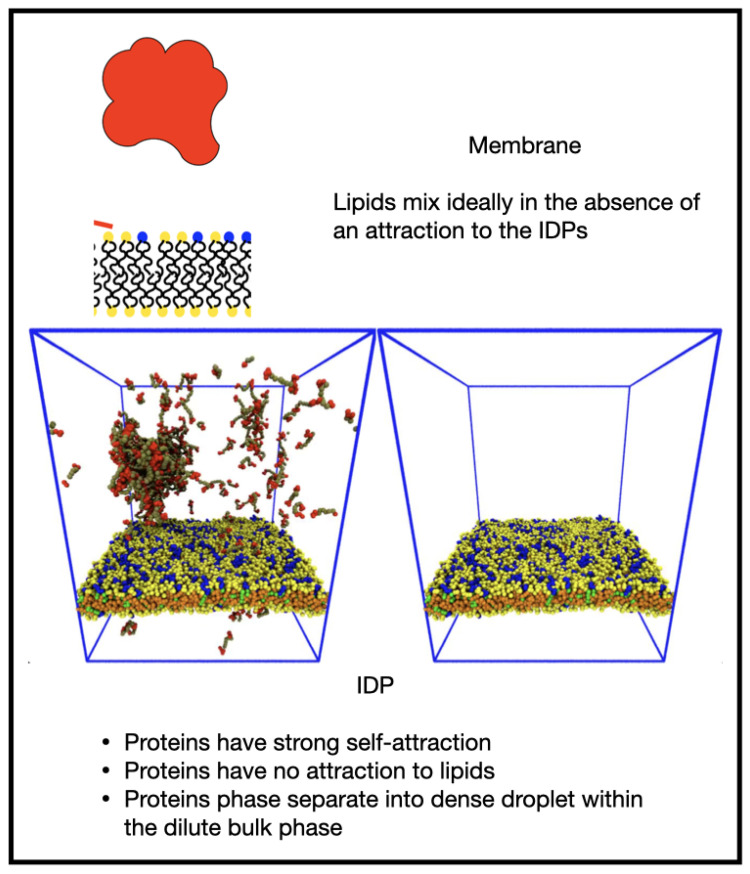
Increasing the IDP self-attraction with no attraction to the membrane leads to a dense protein droplet within a dilute phase, and leaves the membrane well mixed.

Increasing $\varepsilon_{EE}$ while keeping the $\varepsilon_{EM}$ zero led to bulk phase separation of the IDPs without influencing the membrane, which remained well mixed (Figure 5 and Supplementary Video S2). The value of $\varepsilon_{EE}=0.68$ is not very strong, leading to the droplet being in equilibrium with the dilute phase of IDPs dispersed in the solvent.

**Figure 6:**
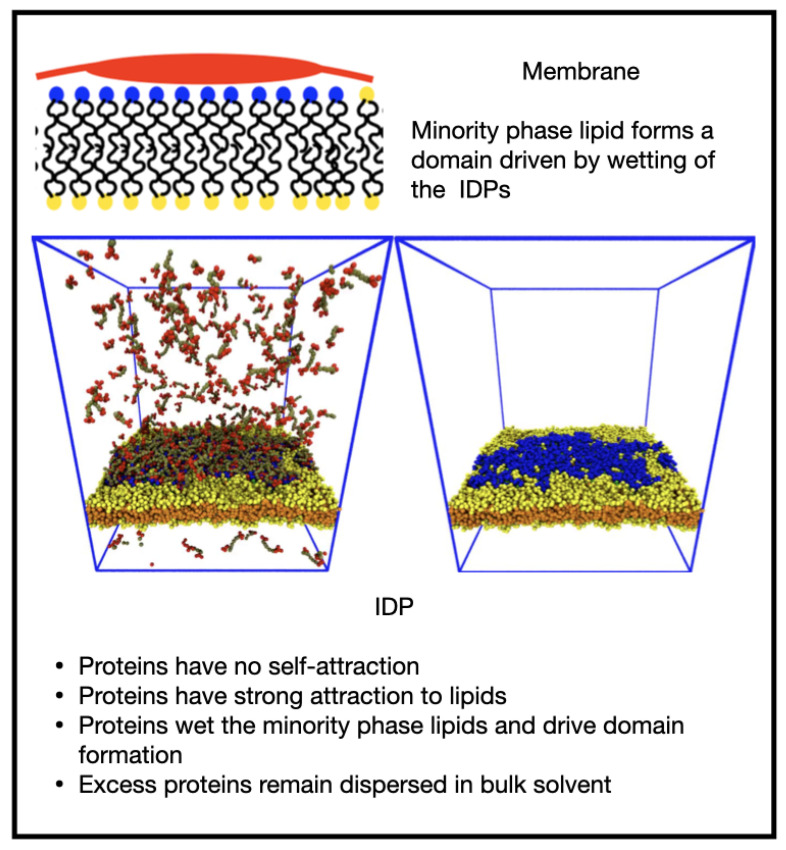
Increasing the IDP–membrane attraction in the absence of any IDP self-attraction leads to wetting of a domain formed of the minority phase lipids.

Increasing $\varepsilon_{EM}=0.8$ while keeping $\varepsilon_{EE}$ zero caused the IDPs to wet the membrane, forming a thin layer as their endcaps bind to the lipid headgroups (Figure 6 and Supplementary Video S3). For the chosen fraction of minority phase lipid, the IDPs were able to saturate the domain leaving some excess IDPs in the solvent. It is interesting to note that the wetting domain formed as the result of the adsorption of the IDPs to the minority lipid headgroups because the IDPs have no tendency to phase separate. The short B_6_ IDPs keep the lipids adsorbed to their endcaps in close proximity. When two or more such adsorbed IDPs approach by diffusion, the thermal fluctuations of the IDPs lead to their mixing between the lipids. This caused the IDPs to effectively connect all the minority lipids into a large domain instead of independent small clusters. However, the wetted domain was non-circular, indicating it has a low line tension, and small patches of lipid occasionally detach, taking a few IDPs with them before reattaching. We note that although the minor lipid species comprises only 16% of the total number of lipids, they are all in the upper monolayer and hence can occupy an area up to 32% of the monolayer’s area.

**Figure 7:**
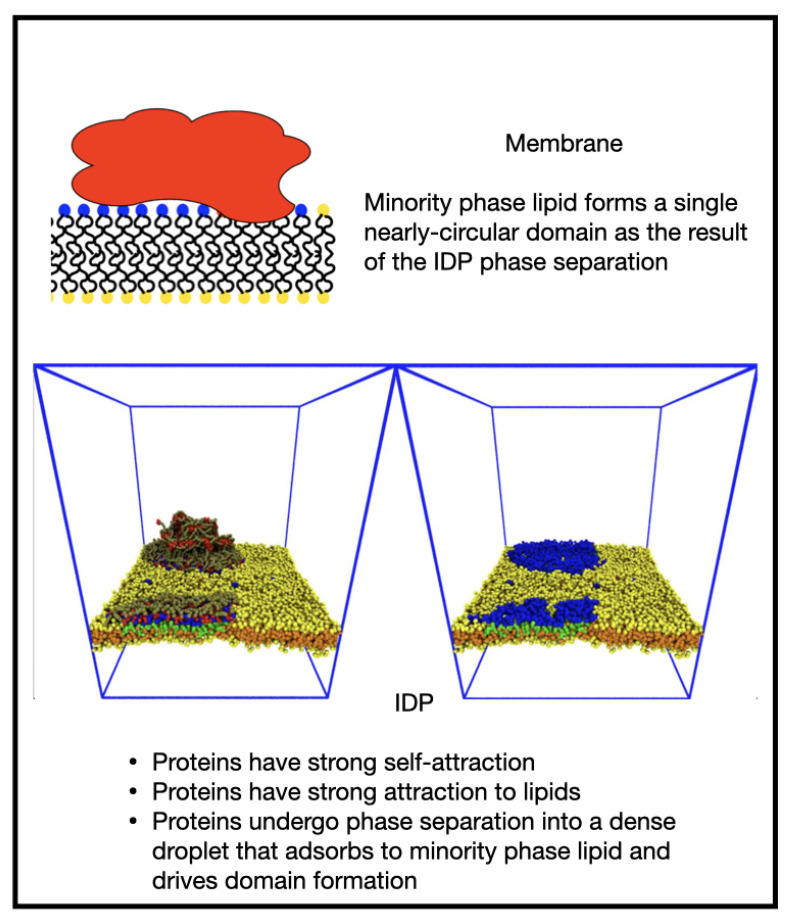
When both IDP self-attraction and the IDP–membrane attraction are strong, the proteins undergo phase separation into a dense droplet that adsorbs to the minority phase lipids driving domain formation but still protrudes into bulk solvent.

Finally, increasing both affinities to $\varepsilon_{EE}=\varepsilon_{EM}=0.8$ led to the formation of a bulk droplet of IDP and wetting of a single domain in the membrane. After diffusing for some time, the droplet adsorbed to the domain, but at the selected concentration, it protruded from the membrane, forming a membrane-associated dense phase. (Figure 7 and Supplementary Video S4). The domain fluctuated around a circular shape, indicating the existence of a fairly high line tension. Small numbers of majority phase lipid sometimes diffused through the domain as seen in the movie Supplementary Video S4.

**Figure 8:**
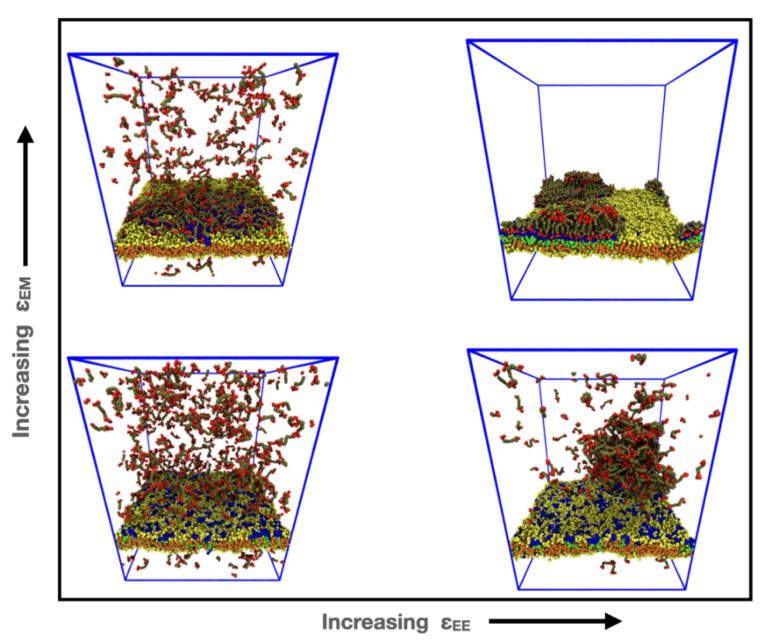
Morphology diagram in the ($\varepsilon_{EE},\;\varepsilon_{EM})$ plane showing the equilibrium states of the IDPs and membrane, as their relative interactions are varied.

Our results for the equilibrium states of the system are summarized in Figure 8 as a two-dimensional morphology diagram with the endcap self-attraction $\varepsilon_{EE}$ along the abscissa and the endcap–lipid headgroup $\varepsilon_{EM}$ along the ordinate. We reemphasize that there was no attraction between the majority and minority phase lipids, and the observed domains were created only by the influence of the IDPs. At the origin, there was no attraction between the IDPs and the membrane nor between themselves, and the membrane was well mixed with the IDPs dispersed in bulk solvent (invisible for clarity). Increasing the IDP self-attraction $\varepsilon_{EE}$ while keeping their attraction to the membrane $\varepsilon_{EM}$ low (bottom right) led to their bulk phase separation while the membrane is well mixed. Increasing the IDP attraction for the membrane $\varepsilon_{EM}$ while it has no self-attraction (top left) led to wetting of the membrane until the minority phase lipids are covered and excess IDPs remain in the bulk. When both interactions were high (top right), the IDPs drove domain formation in the membrane and adsorbed to it forming a droplet that projects into the solvent.

### 3.2. Reversible Coupling of Phase Separation and Domain Formation

**Figure 9:**
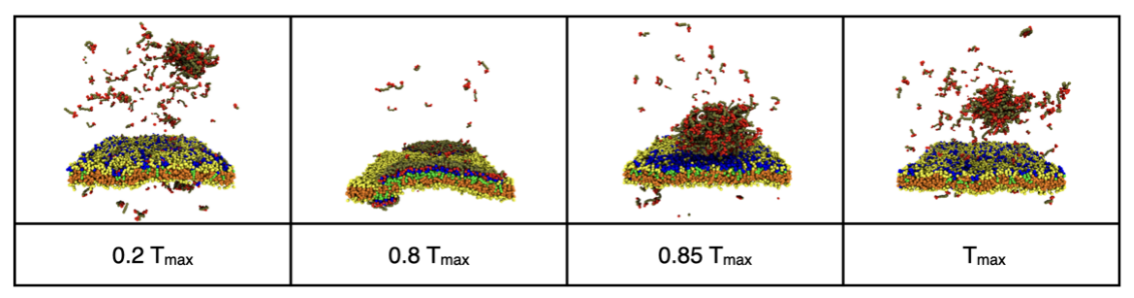
Reversible dissolution of the bulk IDP droplet and wetting of the membrane induced by toggling the interaction of the endcaps (red beads) with the minority phase lipid headgroups (blue beads.).

Membrane domains frequently form transiently in response to cellular signals [32,35]. We observed a reversible transition between a phase separated droplet in the bulk solvent and membrane wetting that was induced by turning on and off the attraction of the IDPs to the minority lipids. Figure 9 shows snapshots from a simulation of 494 IDPs and 395/1978 minority and majority phase lipids, respectively. The timing of the snapshots is given as a fraction of the total simulation time of T_max_ = 3 × 10^6^ time steps. The IDPs were initially neutral towards the minority phase lipids ($\varepsilon_{EM}=0$), but had a self-attraction ($\varepsilon_{EE}=0.68$) that caused them to phase separate into a droplet in the bulk (see Supplementary Video S5). At time 0.2 T_max_, a strong attraction ($\varepsilon_{EM}=0.8$) of the IDP endcaps to the minority lipids was turned on. As the IDPs adsorbed to the minority lipids and drove them into a single domain, the droplet dissolved because the bulk IDP concentration fell below that at which it is in equilibrium with its dilute phase. The IDP/lipid attraction was removed at time 0.85 T_max_ and the IDPs left the membrane and reassembled in the bulk while the membrane rapidly returned to being well mixed. The time required for the membrane to return to a well-mixed state and the IDPs to reassemble in the bulk was much shorter than that for the wetting domain to form.

### 3.3. Increasing IDP Length Results in Patchy Domains

**Figure 10:**
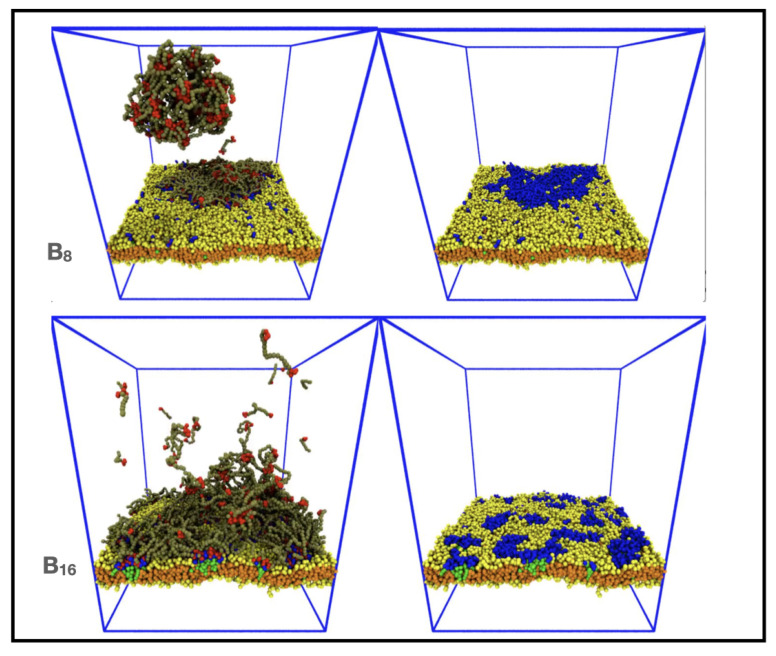
Domains formed from B_8_ (upper panel) and B_16_ (lower panel) IDPs become increasingly patchy and disconnected for strong affinities $\varepsilon_{EE}=\varepsilon_{EM}=0.8$ as the backbone length increases.

We next explored how the backbone length of the IDPs affected the domain morphology. We recall that IDPs with a short backbone B_6_ and high affinities ($\varepsilon_{EE}=\varepsilon_{EM}=0.8$) adsorb to the membrane forming a circular domain that shows small shape fluctuations, indicating a significant line tension (see Figure 7 and Supplementary Video S4). The upper panel of Figure 10 (and Supplementary Video S6) shows that the equilibrium state of a system containing 396 IDPs of the type B_8_ and 396/1982 minority and majority phase lipids, respectively, also contained a single domain, but it contained small patches of the majority lipid and its non-circular shape indicated a lower line tension. On further increasing the IDP backbone length to B_16_ (lower panel of Figure 10 and Supplementary Video S7), we found that the conformationally-fluctuating IDPs bridged multiple, small lipid clusters, but no domain was formed. This simulation contained 296 IDPs and 395/1975 minority and majority phase lipids, respectively, to reduce the crowding effect of the longer IDPs. We hypothesize that the domain formed by short IDPs is broken up by the conformational entropy of the longer IDP backbones. Previous work has shown that B_16_ IDPs assemble into a structured network when they phase separate in the bulk solvent with a spatial structure whose length scale is selected by the length of the IDPs [55]. When these associated IDPs adsorb to the minority lipid headgroups, they maintain this structure which keeps the adsorbed minority lipids farther apart and unable to form a single domain. 

### 3.4. Clustering of Minority Lipids by IDP–Linker Attraction

IDPs are over-represented in cellular signaling and regulatory pathways [63], and are involved in clustering of transmembrane receptors [32]. The cytoplasmic domain of transmembrane receptors plays an important role in their phase separation. One example is the activation of T cell receptors in the immune system, which has been reconstituted in model membranes and leads to formation of a droplet of IDPs associating with the membrane-bound receptors [34]. In addition, the phase separation of IDPs in neurons localizes receptors in the postsynaptic density and vesicles in the pre-synaptic zone [64,65].

**Figure 11:**
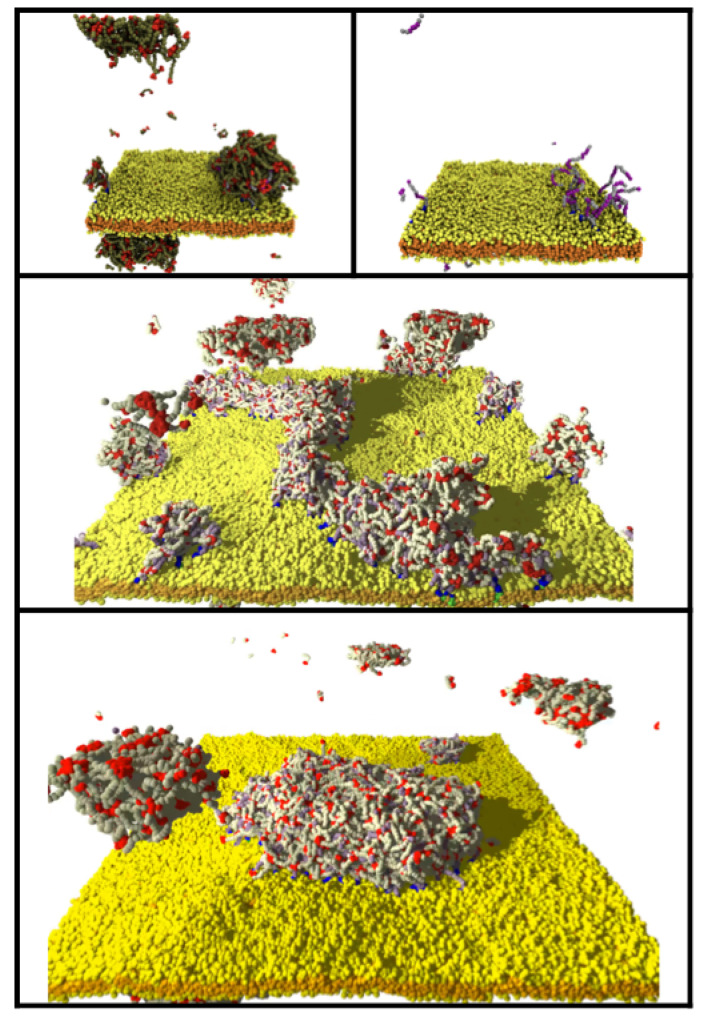
(**Top**) Clustering of linker–lipids driven by the phase separation of 395 IDPs (green with red endcaps) in the smaller system with OSPREY-DPD. (**Middle**, **Bottom**) Intermediate and final states of 1260 IDPs (white with red endcaps) in the larger system simulated by POETS-DPD.

To explore the ability of phase-separating IDPs to drive lipid domain formation via binding to a flexible linker, we attached a hydrophilic linker to the minority lipid headgroup and placed several sticky domains along it that are attracted to the IDP endcaps. The addition of the linkers introduces (at least) an additional three parameters: the number and affinity of the sticky domains, and their separation along the linker. For simplicity, we used a linear sequence of four sticky domains (of four S beads each) separated by inert regions (of four L beads each). The inert and sticky linker domains are hydrophilic and have no net attraction. We defined the dimensionless strength of the attraction between the IDP endcaps and the sticky domains ($\varepsilon_{ES}$) similarly to the endcaps, but used the linker–linker sticky domain conservative parameter as the baseline: $\varepsilon_{ES}=\left(a_{SS}-a_{ES}\right)/a_{SS}$. To avoid artifacts of the linkers prematurely clustering due to their initial configuration, we set the endcap–linker interaction to $\varepsilon_{ES}=0$ ($a_{SS}=a_{ES}=30$), and equilibrated the system for 250,000 time steps allowing the minority lipids to diffuse freely across the membrane. Then the endcap self-attraction and endcap–linker attraction were set to a strong value, $\varepsilon_{EE}=\varepsilon_{ES}=0.83$. Figure 11 shows the effects of 9 linker–lipids present in the membrane. As the IDPs phase separated, they bound to the linkers and slowly merged into a large droplet that drew the minority lipid/linkers into a domain (Figure 11, top panel, and Supplementary Video S8. 

The above results were generated using the OSPREY-DPD code in a simulation box of size 40 × 40 × 48 (*d_0_*)^3^. In Figure 11 (and Supplementary Video S9), we compare the case of LLPS-driven domain formation from Figure 7 with those generated using the POETS-DPD code in the larger simulation box 100 × 100 × 48 (*d_0_*)^3^. The upper panel shows the equilibrium state in the small simulation box. We found that as few as 7 linker–lipids can bind a droplet of 395 IDPs whose size is much larger than the clustered linkers. Two linkers flip-flopped from the upper leaflet as a consequence of the initial state and were able to bind sufficient IDPs to form a second domain visible in the lower left (and top left as it is connected across the periodic boundaries). The middle and lower panels of Figure 11 show two views of the equivalent simulation executed with POETS-DPD that contains a membrane that is 100 nm in linear dimension and contains 1260 IDPs and 2520 linker–lipids. The same clustering of the larger number of IDPs to the larger number of linker–lipids results in a similar droplet bound to the membrane (cp. Supplementary Videos S8 and S9).

## 4. Discussion

The increasing number of biomolecular condensates discovered in cells and the ubiquity of membrane-bound organelles suggests that interactions between them are likely to be important for cellular physiology. They also provide an interesting conceptual system for exploring how the two-dimensional nature of a membrane interacts with three-dimensional droplets. An example is provided by the phase separation of multivalent IDPs coupled to transmembrane signaling proteins in immune cell signaling [11,35]. We used coarse-grained molecular simulations to explore the morphologies adopted by a dilute phase of IDPs near a two-component lipid membrane. The high computational cost of such simulations drove this collaboration between biophysicists and the developers of novel computer hardware to explore how coarse-grained simulations on much larger scales can be accelerated and yet consume less electricity.

Our biological results are summarized in the morphology diagram in Figure 8, where we explored the relationship between the molecular properties of the constituent molecules and their equilibrium morphological phase behavior. Two non-trivial outcomes were observed. First, if the IDP endcaps were more strongly attracted to the minority phase lipid headgroups than to each other, they wet the membrane. If the length of the IDPs is short, this drove the minority lipids into a single large domain, while longer IDPs adsorbed via multiple, small, discrete clusters. When the IDP self-interaction and their interaction with the minority lipid headgroup (or with sticky linkers attached to the headgroup) were both sufficiently large, bulk LLPS occurred and drove formation of a single membrane domain.

Most of the preceding results were obtained from small systems with around 2400 lipids in the membrane and several hundred IDPs, comprising 230,000 particles in total. Each simulation required 14 cpu-days on a single core of a Ryzen Threadripper 3970X 4.5 GHz desktop machine. It is tedious and unfeasible to carry out hundreds of these simulations to explore exhaustively the parameter space of such complex models. In addition, the ~70 simulations we performed consumed an environmentally-unsustainable amount of electricity. We therefore performed some larger simulations in a box size 100 × 100 × 48 (*d_0_*)^3^ on a novel computer hardware called POETS that drastically reduces the wall clock time of our DPD simulations. The larger system contains 14,600 lipids and 1260 IDPs, comprising 1,400,000 particles in total, but requires only 3 days to run the same number of time-steps as the smaller case.

The research goal of our collaboration of biophysicists and computer engineers is to ensure that the new POETS algorithms and hardware agree with the established DPD codebase (for which many published results exist); that they can handle real-world problems that practitioners want to solve; and that they are highly scalable. Initially, we focused on matching the performance of single-node systems such as shared-memory multi-core CPUs and individual GPUs.

Providing direct comparisons between disparate hardware/software systems is very difficult. For example, it is often unclear how much systems actually cost (buying 1 unit is different from buying 1000), what their power consumption is (should cooling costs be included?), and whether computations are equivalent (is single-precision the same as double-precision?). Table 2 shows indicative metrics for how different systems behave in practice, based on published figures and (where possible) our own experiments. We characterized them in terms of one compute-node, where a node is a multi-core CPU, GPU plus support CPU, or an FPGA card for POETS, with power consumption quoted per node. The performance was measured in total bead-steps per second across all nodes, which is simply the total number of beads multiplied by the total number of simulation time steps, divided by the execution time. CPU figures are based on our own measurements, while GPU figures are courtesy of Seaton [66].

The figures for “POETS Gen1” are the performance attained in this work, which is the measured performance of the hardware performing the large-scale simulations described in this paper. We clearly exceeded the performance of the single-core Osprey-DPD simulator used for the parameter exploration, which is our baseline comparison. It is also slightly faster than a high-performance 32-core machine in our HPC cluster when using multi-core LAMMPS, and about the same performance as a single high-performance GPU. However, we can see that the performance per watt was relatively weak—similar to a single-threaded implementation running on a CPU. This is a consequence of two effects: First, the hardware we used is the first generation, which is based on old FPGAs from 2011; second, we have so far focused on correctness and useability, and have spent little time on performance optimization.

Overall, we achieved the research goals of the collaboration in producing a new hardware system and algorithms that:

- Produce reliable DPD results (agree with published DPD implementation) [53];
- Are useful for practitioners;
- Are competitive in performance with existing single-node systems.

We are currently assembling the second generation of POETS hardware (POETS Gen2), which is substantially more powerful as it is built on more recent FPGAs. This increases the number of cores by a factor of 10, and the floating-point performance per core by a factor of 4, so we have included in Table 2 estimated figures for the POETS-DPD implementation running on this new hardware. At this point, the performance and efficiency start to become competitive with multi-GPU systems, even without additional tuning and algorithmic optimizations. For example, we have already found that dynamic load-balancing within the POETS hardware dramatically improves simulation rate. 

Looking further ahead, we are actively exploring new algorithms and optimizations which substantially improve the performance per FPGA node. In particular, adding custom instructions designed to support DPD calculations will reduce the number of instructions per bead interaction by a factor of 10. We included tentative estimates for the eventual performance we are targeting using the hardware currently being assembled, based on reasonable extrapolations. Clearly more research is needed, and this level of performance is an aspirational goal, but having demonstrated that the basic idea works and is useful we have confidence that this target is worth pursuing. 

## 5. Conclusions

We live in interesting times: The power of computer systems is increasing year-by-year, bringing biological fidelity at increasing length scales and ever longer time periods (almost) within our grasp. Existing technologies such as multi-core, MPI, and GPUs provide one way of scaling to larger simulations, but it is increasingly important to reduce the financial and environmental costs of the simulations. Exotic architectures with custom hardware are another way, but it is unclear whether they work for real-world problems, or even if they give the correct results.

The principal goal of this paper was to identify the equilibrium morphologies of a model of phase separating proteins near a two-component membrane using coarse-grained simulations. But a secondary goal was to demonstrate how biophysical simulation needs can drive inter-disciplinary research that yields faster and cheaper simulations. By embedding the development of a new hardware simulation platform into the scientific process, we create a feedback loop between the computational modeling pull and the computer technology push that ensures the hardware advances work in practice—not just in theory. The results presented in this paper were generated on distinct compute platforms (Single core: Ryzon ThreadRipper 3970X; 4.5 GHz x86 32-core desktop; POETS machine: 49000 RISC-V threads): Each platform (running different codebases) provides consistent results, which is an important and often overlooked aspect of simulation-based research.

Our final aim was to illuminate the path towards using event-based computing to extend computational biophysics into more realistic arenas of membranes in health and disease. Extensions to our work could follow several interesting routes. The assumption of ideal mixing of the lipid species in the membrane could be relaxed to explore the converse effect of domain formation on the bulk phase separation. The model IDPs could also be more closely modeled on real proteins by adding multiple, punctate binding sites, or a mixture of IDPs could be included. However, increasing the biological fidelity creates more complex models with more parameters, making a systematic exploration of their behaviour challenging on conventional architectures. The POETs computer framework introduced here accelerates DPD simulations sufficiently that the exploration of high-dimensional parameter space models is feasible while simultaneously reducing the energy consumed. As simulations are increasingly used to augment wet-lab experiments, this will become an increasingly important route to sustainable science.

## Figures and Tables

**Table 1:** Bead–bead conservative force parameters $a_{ij}$ (in units of $k_{B}T/d_{0}$) for all bead types. The table is symmetric. The parameters $a_{EE}$, $a_{EM}$, and $a_{ES}\;$ specify the attraction between the IDP endcaps and themselves, the minority lipid headgroup, and the sticky domains in the linkers respectively as described in the text.

$a_{ij}$	W	E	B	H_A_	T_A_	H_B_	T_B_	L	S
W	25								
E	25	*a* *_EE_*							
B	23	25	25						
H_A_	30	30	30	30					
T_A_	75	35	35	35	10				
H_B_	30	*a* *_EM_*	30	30	35	30			
T_B_	75	35	35	35	10	35	10		
L	30	30	30	30	35	30	35	30	
S	30	*a* *_ES_*	30	30	35	30	35	30	30

**Table 2:** Indicative performance of distinct compute platforms. Year: year hardware platform was introduced; Watts: power consumption of one node under load; Nodes: # compute nodes; Perf: performance in millions of bead-steps per second; Perf/Watt: performance per watt of electricity consumed.

System	Year	Software	Watts	Nodes	Perf/10^6^	Perf/Watt
CPUx1	2020	Osprey-DPD	250	1	0.5	2000
CPUx32	2020	LAMMPS	500	1	19.0	38,000
GPUx1	2016	DL-MESO	400	1	23.7	59,195
GPUx8	2016	DL-MESO	400	32	285.7	22,321
POETS Gen1	2011	POETS-DPD	200	48	24.0	2500
POETS Gen2	2022	POETS-DPD	250	48	360.0	30,000
Target parameters for next generation POETS system:						
POETS Gen3	2025	POETS-DPD	250	48	3600.0	300,000

## Data Availability

Simulation datasets are available on reasonable request to the corresponding author.

## Supplementary Materials

The following are available online at https://www.mdpi.com/article/10.3390/membranes12010017/s1, Video SM1: Dispersed B_6_ IDPs and well-mixed membrane ($\varepsilon_{EE}=\varepsilon_{EM}=0$), Video S2: Bulk LLPS of B_6_ IDPs and well-mixed membrane ($\varepsilon_{EE}=0.68,\;\varepsilon_{EM}=0$), Video S3: Wetting of membrane by B_6_ IDPs drives domain formation ($\varepsilon_{EE}=0,\;\varepsilon_{EM}=0.8$), Video S4: LLPS of B_6_ IDPs drives domain formation ($\varepsilon_{EE}=\varepsilon_{EM}=0.8$), Video S5: Reversible LLPS and domain formation of B_6_ IDPs ($\varepsilon_{EE}=0.68,\;\varepsilon_{EM}=0.8$), Video S6: LLPS of B_8_ IDPs creates patchy domains ($\varepsilon_{EE}=\varepsilon_{EM}=0.8$), Video S7: LLPS of B_16_ IDPs creates small lipid clusters ($\varepsilon_{EE}=\varepsilon_{EM}=0.8$), Video S8: LLPS of B_8_ IDPs and linker binding drives domain formation ($\varepsilon_{EE}=\varepsilon_{ES}=0.83$), Video S9: LLPS of B_8_ IDPs and linker binding drives domain formation in (100 nm)^3^ box ($\varepsilon_{EE}=\varepsilon_{ES}=0.83$).
